# Extraordinary Number of Convulsions in a Child

**Published:** 1837

**Authors:** Wolcott Richards

**Affiliations:** Cincinnati


### Art VI.—Exrtaordinary Number of Convulsions in a Child

Communicated by Wolcott Richards, M. D., of Cincinnati.

J. H. P. was born November 15th, 1835. From the time of his birth he suffered from constipation of the bowels, attended by occasional paroxysms of colic. So obstinate was the cosiveness, that either enemas or suppositories were required almost daily. With this exception, he appeared to enjoy good health; and continued to thrive till the 6th of Feb.; when he was siezed with a catarrhal affection, manifested by cough, running of the eyes and nose, and quick pulse. Skin dry excepting about the head, which was frequently bedewed with perspiration. I prescribed a purgative, with expectorants, and diaphoretics.

Feb. 7th. Febrile symptoms the same; respiration somewhat laborious; considerable coma, so that it was with some difficulty the child could be roused. Gave an emetic, which after severalr epetitions, operated freely upon the stomach and bowels; this was followed by small doses of calomel and ipicac frequently repeated.

Feb. 8th. Comatose state removed; fever diminished; cough lesened. Ordered oleaginous mixture.

Feb. 9. Appeared as well as usual.

Feb. 12. Was sent for in the night. Found him in convulsions. He had just been taken from a warm bath. Ordered an enema containing asafœtida; applied sinapisms to the lower extremities, and cold applications to the head. So soon as he could swallow, gave 5 grs. of cal. followed by oleaginous mixture.

The convulsions continued at short intervals till the bowels were freely operated upon.

Feb. 13th. Somewhat comatose; pulse slow, discharges from the bowels light green color, with much mucus. Prescribed small doses of cal. and ipecac alternately, with oleaginous mixture.

Feb. 14th. Symptoms better. Appeared much more lively, nursed freely. Secretions unimproved. Continued medicines.

Feb. 15th. Convulsions returned with increased violence. From this time till the day of his death (which occured on the 9th of March, at the age of nearly four months) he had many convulsions daily. Sometimes forty or fifty in twenty-four hours. During the intervals (when they were sufficiently extended for him to recover from the immediate effects of the paroxysm) the only morbid symptoms which presented themselves, were green and slimy evacuations from the bowels, (which continued from the beginning, resisting all remedies.)

A slow pulse, less than 70 in a minute, and at times, pain or uneasiness manifested by screaming.

During the convulsions, and immediately thereafter, there were strabismus, and dilatation of the pupil. But the strabismus disappeared in a few minutes, and the restored mobility of the iris indicated the usual sensibility of the retina to light. In this condition he lingered about three weeks, having had during that period, at the lowest calculation, upwards of 500 distinct convulsions.

A *post mortem* examination was made by Dr. Gross, professor of Pathological Anatomy in the Cincinnati College, in presence of several medical gentlemen; the results of which are correctly stated in his own words, as follows:—"In this case I made a careful examination of the brain and spinal cord, and the thoracic, abdominal and pelvic viscera, without finding the slightest lesion of any kind. There was no sign whatever of cerebral disease, such as inflammation, softening, induration, or serous or bloody effusion; the mucus membrane of the stomach and bowels was perfectly sound throughout; there was no lesion of the glands of Brunner or of Peyer; the mesenteric ganglia were not enlarged; and in short, there was no derangement of structure of any kind, by which the convulsions or the child's death can be explained."

*March 9th*, 1837.

### Art VII.—Fatal Case of Concealed Strangulated Hernia. Contributed by William S. Ridgely, M. D.

In the month of January, 1837, I was desired to visit Mrs R., aged 45 years, whom I found in a state of costiveness with vomiting. For the three preceding days, she had not been able to procure an evacuation, although she had taken freely of several active cathartics. She had no pain of the abdomen, except an occasional slight paroxysm in the epigastric region. She had no fever, her pulse being calm, and her skin rather cool than otherwise. I prescribed several doses of cathartic medicine, combined at first with large portions of Extract of Hyosciamus. In about 24 hours she had a copious alvine evacuation, which for a while greatly improved her feelings; but her vomiting still continued, and only ceased a short time before her death, which occurred on the fourth day after my first visit. After the evacuation of which I

have spoken, as others could not be procured, I bled her to the amount of a pound, which produced some composure with a protracted tendency to syncope. The blood exhibited a trace of siziveness. Her abdomen was somewhat tender on pressure towards the close of the disease, but neither swollen nor hard. She was carefully examined for hernia by Dr. Drake and myself, but no tumor was perceptible. For twelve hours before her dissolution, her anxiety and restlessness were very great, and her pulse became extremely weak, while her intellectual functions remained unimpaired.

After death I requested professor Gross to assist me in making an examination of the morbid appearances, and the result has been correctly stated by him in the following note.

The protrusion, which was about the size of a large almond, was formed by a portion of the ilium, scarcely a third of an inch long, which had found its way into the internal ring.—The structure itself seemed to have been caused by a fold of peritoneum, stretched tightly across the upper border of the ring. That this was the case is sufficiently proved by the fact, that the structure still remains in the preparation, although it consists solely of intestine and peritoneum. The portion of bowel between the ring and the ileo-cœcal valve, was about four feet in length, quite empty and contracted, and without any sign of inflammation. Above this point, the small intestine was greatly distended with gas and fœcal matter, and bore all the marks of severe inflammation, the redness, which was of a violet hue, occurring in small patches in different parts of the caliber of the tube. The serous covering of the bowel was studded with numerous globules of lymph; and the incarcerated portion was in a state of incipient gangrene, being of a light brownish color. There was no fibrin thrown around the margins of the ring; and the structure was so slight that the strangulated gut was easily withdrawn from its unnatural situation. The colon was sound, but extremely contracted throughout the greater portion of its extent, being scarcely large enough to admit the little finger.

Her rectum was in the same condition. All the other abdominal organs, as well as those of the thorax, were in a normal state. The peritoneum was natural, excepting immediately round the seat of the structure, where it was very vascular and inflamed. *No external tumor whatever could be perceived in the region of the groin.*

### Art. VIII.—Abstract of Meteorological Observations.

*Abstract of Meteorological Observations for the year* 1836, *taken at the* Woodward College, *Cincinnati: Lat.* 39° 6 *N. Long* 84° 27 W. By Joseph Ray, M. D., Professor of Mathematics and Natural Philosophy.

| | |
|---|---|
| Mean temperature of the year, | 51° 637 |
| Maximum height of thermometer July 12th and 23d, | 99.0 |
| Minimum do. do. Feb. 3d, | 7.0 |
| Range of thermometer, | 10.60 |
| Warmest day in the year, July 12th, | 82° 8 |
| Coldest day in the year, Feb. 1st, | 3.1 |
| Maximum height of barometer, March 25th, and Dec. 22d, | 29.82 inches. |
| Minimum do. do. Dec. 13, | 28.66 |
| Mean do. do. | 29.3451 |
| Perpendicular depth of rain and melted snow (inches) | 57.39 |
| Dryest month in the year, | June. |
| Wetest month in the year, | May. |
| Clear and fair days, | 134 |
| Variable do. | 116 |
| Cloudy do. | 116 |

Wind, N. 26 days—N. E. 63½ days—E. 24½—S. E. 28 days—S. 15 days—S. W. 74½ days—W. 80½—N. W. 54 days.

The Ohio river at this city, was frozen over from Feb. 1st to Feb. 14th. The mean temperature both of this year and 1835 was considerably below the mean annual temperature of a series of years. I find that Dr. Hildreth, of Marietta, makes the mean temperature of 1835 4° below the mean of a series of years at that place. The greatest depression of temperature in two years took place in Feb.

1835, yet both January and March 1836, were colder than the same months in 1835; without taking into account the corresponding mean temperature of the other months of those years this will in some measure account for their equality of mean temperature. By a comparison of observations in different sections of the union, it is obvious that the year 1835 was remarkable for the low mean temperature, and as far as my observations enable me to judge, the year 1836, though not so remarkable for any single depression of temperature, on the whole partakes of the same character.

### Art. IX.—Brief Notices of the Weather and Diseases in Cincinnati, in the months of January, February and March, 1837.

In our last number we stated in general terms the character of the diseases, prevalent in the latter part of the year 1836. It was decidedly phlogistic. In January and February, the governing malady was scarlatina anginosa, of which it may be well to mention a few of the leading traits. In no instance did we see ulceration of the fauces, though in every case there was mucous inflammation. This inflammation was not limited to the membrane, but extended into the solid gangliæ and the surrounding cellular tissue. Thus the tonsils, the salivary glands, and the lymphatic ganglia of the neck were engorged and tumefied. A great deal of ear ache frequently occurred, followed in several cases by a purulent discharge from the meatus auditorious.—The fever was not of the highest inflammatory tone; but approached more to the type, which in the nosology of Cullen, is denominated synochus. We did not however witness any example of its degenerating into typhus. Notwithstanding its apparent mildness, cases now and then occurred, of what, in the common parlance of the profession, are called congestive; that is, in which the blood accumulated in the central organs, cranial, thoracic, and abdominal. This pathological state was in several patients, productive of violent vomiting and diarrhœa, with extreme prostration.

The lancet has not been much employed through the winter in scarlatina. The common remedies have been emetics and cathartics. The prescription upon which we ourselves most relied, was the antimonial powder of Dr. Rush; and the *cool* affusion, which we preferred to immersion. In advanced stages, the sulphate of quinine, dissolved in water, acidulated with elixer vitriol was sometimes found beneficial. A medical friend spoke to us in high terms of its efficacy in several cases.

We must confess, however, that we are not conscious of having done much good by any plan of treatment; and in reviewing our practice and its results in past times, we begin seriously to doubt, whether any thing we have ever employed, was of much service. Indeed, to speak out fully on the subject, we are disposed to think that no method of treatment hitherto adopted in scarlatina, has materially increased the number of recoveries. We have seen patients die after blood-letting had been carried to syncope; and have quite a conviction, that the lancet has not often been beneficial. A hot atmosphere is no doubt pernicious; but on the other hand, the efficacy of one decidedly cold, may well be questioned.

The mumps have prevailed in connection with scarlatina; and very often appeared to succeed that disease, in the same patient. We say *appeared*, because in several cases that fell under our observation, it was difficult to decide, whether the swelling and suppurative inflammation of the parotid glands, arose from the poison of scarlatina or mumps. In two patients of the same family, suppuration of these glands followed scarlatina, and required to be opened; in one, the discharge was thin bloody and sanious—in the other, of a healthy, purulent character: the former died, the latter recovered.

Both these children were leeched extensively over the inflamed parts, without dispersing the congestion. We have not through the winter, met with a case of metastasis, to the teses or mammæ.

Within the period of which we are treating, a number of mild cases of varioloid or virecella have occurred.

The measles are now beginning to supplant the scarlatina. As yet the disease is of the mildest kind. Like the scarlet fever, it is, however, in some cases followed by parotideal inflammation.

From these hasty notices it will be seen, that we have recently been visited by no less than four of those epidemics, which are reputed to be contagious, and respectively affect the individual but once. Such a combination is not unprecedented, but but by no means common.

With the exception of these maladies, the people of Cincinnati have not been sickly beyond the ordinary degree.

The winter has not been at any time intensely cold, but protracted; and now, March 31st, vegetation is unusually backward.

### Art. X.—Consultation Letters.

Physicians who reside in cities, and earn a reputation that is bruited abroad to any distance, especially if they are known as teachers or writers, are very often consulted by letter on difficult cases. These letters are sometimes written by the patients themselves; at other times, by their physicians. The reports and statements made in them are often exceedingly imperfect, and impose on the physicians and surgeons to whom they are sent, a most difficult task. Those who write them should be aware of the necessity of making them ample in their details, that they may furnish all the data that are necessary to a correct decision. In a great majority of these applications, no compensation is remitted to the person to whom they are sent, although, if the patient came personally, he would expect to pay for the advice he received. Such omissions are unjust, and he who commits them should expect to have his communcations neglected. It was, as we have understood, the custom of Dr. Rush, to require a ten dollar bill to be enclosed in every letter of consultation; a practice that should not be condemned as sordid; inasmuch, as it signifies not in what manner a physician is employed. He must live by his profession, and it should not be asked of him, except for the indigent, to give gratuitous advice.

### Art. XI.—Progress of Quackery.

A most barefaced attempt was lately made by the Steam and Botanical doctors of our state, to get themselves incorporated by the Legislature. The bill passed in the Senate, but was lost in the House of Representatives. The application will no doubt be revived next winter; but it may be hoped that the good sense of the community (concentrated as it is in the General Assembly) will again defeat them. It was bad enough, in all conscience, to repeal the law which discountenanced the practice of empyricism; but to proceed afterwards to the building up of a College for the manufacture of steam, botanical and Indian doctors, would be a step beyond what any of the United States have taken. The friends of science and the *real* friends of the people, should labor to ward off the calamity, and preserve our statute book from so foul a stain.

### Art. XII.—Discourses and Lectures.

Several gentlemen have favored us with copies of their discourses and lectures on various subjects. We have not had time to look over the whole; but our next number will show that we shall not overlook any, which rise to the grade of tolerable.

